# Biological sex does not impact intrinsic mitochondrial respiration supported by complexes I and II in human skeletal muscle

**DOI:** 10.1113/EP092551

**Published:** 2025-03-26

**Authors:** Emily J. Ferguson, Lauren J. Pacitti, Justin Bureau, Callum J. Pufahl, Eveline Menezes, Tanner Stokes, Shivam Gandhi, Luca J. Delfinis, Craig A. Simpson, Christopher G. R. Perry, Brendon J. Gurd, Chris McGlory

**Affiliations:** ^1^ School of Kinesiology and Health Studies Queen's University Kingston Ontario Canada; ^2^ School of Kinesiology & Health Science, Muscle Health Research Centre York University Toronto Ontario Canada; ^3^ Department of Medicine Queen's University Kingston Ontario Canada

**Keywords:** aerobic fitness, biological sex, creatine, human skeletal muscle, intrinsic mitochondrial respiration, mitochondrial content

## Abstract

The effect of biological sex on rates of skeletal muscle mitochondrial respiration supported by creatine‐dependent phosphate shuttling was previously unknown. The aim of this investigation was to test the hypothesis that females and males matched for peak oxygen uptake normalized to fat‐free mass would not exhibit differences in rates of mass‐specific and intrinsic mitochondrial respiration in the presence or absence of creatine. Rates of mass‐specific and intrinsic complex I (pyruvate and malate)‐ and complex I+II‐supported, adenosine diphosphate (ADP)‐stimulated mitochondrial respiration in the presence and absence of 20 mM creatine were measured via high‐resolution respirometry. Total, intermyofibrillar and subsarcolemmal mitochondrial volume density were analysed using transmission electron microscopy. Rates of intrinsic mitochondrial respiration were obtained by normalizing mass‐specific respiration rates to total mitochondrial volume density and total electron transport chain subunit protein content. Overall, there was no effect of sex on rates of mass‐specific or intrinsic mitochondrial respiration in the presence or absence of creatine. There was also no effect of sex on total, intermyofibrillar and subsarcolemmal mitochondrial volume density or electron transport chain subunit protein content. Our data demonstrate an overall lack of sex‐based differences in rates of intrinsic complex I‐ and complex I+II‐supported, ADP‐stimulated mitochondrial respiration in the presence or absence of creatine in females and males matched for aerobic fitness. Thus, biological sex per se does not appear to modulate intrinsic skeletal muscle mitochondrial respiration in healthy young adults.

## Abstract (highlights)

- **What is the central question of this study?**
  Mitochondrial–cytosolic energy exchange can occur via creatine‐dependent and ‐independent mechanisms, but whether biological sex impacts rates of intrinsic mitochondrial respiration in the presence of creatine in human skeletal muscle remains unknown.
- **What is the main finding and its importance?**
  Biological sex does not impact mitochondrial volume density or intrinsic mitochondrial respiration supported by creatine‐dependent phosphate shuttling and creatine‐independent ADP diffusion when females and males are matched for peak oxygen uptake normalized to fat‐free mass. Matching females and males for aerobic fitness mitigates sex differences in rates of mitochondrial respiration and mitochondrial content in humans.

## 1 INTRODUCTION

Skeletal muscle mitochondria consume oxygen via mitochondrial respiration, which can be coupled to the regeneration of energy in the form of adenosine triphosphate (ATP) through a process known as oxidative phosphorylation. Rates of skeletal muscle mitochondrial respiration can be assessed in suspensions of isolated mitochondria or permeabilized muscle fibre bundles (PmFBs). However, mitochondrial isolation and muscle fibre permeabilization lead to differences in mitochondrial structure, function and interactions with intracellular structures (Picard et al., 2011). In PmFBs, mitochondrial–cytoskeletal interactions that provide a physical diffusion barrier for mitochondrial substrates, such as adenosine diphosphate (ADP), are preserved (Guzun et al., 2012; Picard et al., 2011) providing an ideal model for studying mitochondrial respiration in conditions that might better reflect the in vivo cytoskeletal environment (Picard et al., 2011).

Given the potential role of sex hormones in substrate metabolism (Tarnopolsky, 2008), the impact of biological sex on skeletal muscle mitochondrial respiration is a focus of intense investigation. Some studies have shown that biological sex may modulate rates of mitochondrial respiration in PmFBs (Miotto et al., 2018; Montero et al., 2018), whereas others have not (Schytz et al., 2024). Discrepant findings among previous investigations could be attributable to several conflating factors, including differences in aerobic fitness between female and male participants. Matching female and male participants for aerobic fitness is important because individuals with higher aerobic fitness exhibit higher mitochondrial respiration rates and mitochondrial content (Jacobs & Lundby, 2013; Schytz et al., 2024; Zoll et al., 2002). Although not all prior studies in PmFBs matched female and male participants for peak oxygen uptake (${\dot{V}}_{O_{2}\mathrm{peak}}$; Miotto et al., 2018), when matched for ${\dot{V}}_{O_{2}\mathrm{peak}}$ normalized to body mass, females demonstrate higher mitochondrial volume density (mito_VD_) and mass‐specific complex I‐supported, ADP‐stimulated respiration rates in PmFBs compared with males (Montero et al., 2018). However, matching females and males for ${\dot{V}}_{O_{2}\mathrm{peak}}$ normalized to body mass might result in discrepancies in aerobic fitness. Females and males differ with respect to body mass, body composition and other physiological factors impacting whole‐body ${\dot{V}}_{O_{2}\mathrm{peak}}$, such as haemoglobin mass (Caswell et al., 2024; Ofenheimer et al., 2020). Females typically have a higher body fat percentage (Ofenheimer et al., 2020), hence less biological tissue contributing to ${\dot{V}}_{O_{2}\mathrm{peak}}$, relative to males. Thus, comparing participants matched for ${\dot{V}}_{O_{2}\mathrm{peak}}$ normalized to fat‐free mass (FFM) is the recommended method to control for differences in fitness between females and males (Tarnopolsky, 2008). To our knowledge, no study has examined rates of mass‐specific and intrinsic skeletal muscle mitochondrial respiration in PmFBs from females and males matched for ${\dot{V}}_{O_{2}\mathrm{peak}}$ normalized to FFM.

Energy transfer between the mitochondria and cytosol occurs via creatine‐dependent and ‐independent mechanisms (Perry et al., 2013). Data generated thus far regarding whether biological sex impacts rates of skeletal muscle mitochondrial respiration in PmFBs have been obtained without the addition of creatine to the respiration media. Experiments conducted in the absence of creatine yield important insight into the function and regulation of creatine‐independent ADP and ATP diffusion alone (Miotto & Holloway, 2016; Ydfors et al., 2016). However, under physiological conditions, skeletal muscle is never devoid of creatine (Perry et al., 2012; Tonkonogi et al., 1998; Walsh et al., 2001; Ydfors et al., 2016), and creatine‐dependent phosphate shuttling, which requires the addition of creatine to the respiration media, is argued to be the more predominant and efficient mitochondrial–cytosolic energy‐exchange mechanism (Perry et al., 2012; Ydfors et al., 2016; Aliev et al., 2011). Thus, it is important to measure rates of respiration both with and without creatine in the respiration media to assess respiration supported by both creatine‐dependent and creatine‐independent mitochondrial–cytosolic energy exchange mechanisms. However, whether females and males matched for ${\dot{V}}_{O_{2}\mathrm{peak}}$ normalized to FFM exhibit different rates of mass‐specific and intrinsic skeletal muscle mitochondrial respiration in both the presence and absence of creatine in PmFBs is unknown.

The purpose of this investigation was to examine sex differences in rates of skeletal muscle mitochondrial respiration in PmFBs from females and males matched for ${\dot{V}}_{O_{2}\mathrm{peak}}$ normalized to FFM in the presence and absence of creatine. Our primary objective was to assess whether females and males matched for aerobic fitness exhibit different rates of mass‐specific and intrinsic complex I‐ and complex I+II‐supported, ADP‐stimulated respiration in the presence and absence of creatine. A secondary objective was to compare total, intermyofibrillar (IMF) and subsarcolemmal (SS) mito_VD_ and electron transport chain (ETC) subunit protein content in females and males matched for aerobic fitness. We hypothesized that females and males matched for aerobic fitness would not demonstrate statistically significant differences in rates of mass‐specific and intrinsic respiration in both the presence and absence of 20 mM creatine. We also hypothesized that females and males matched for ${\dot{V}}_{O_{2}\mathrm{peak}}$ normalized to FFM would not exhibit statistically significant differences in total, IMF and SS mito_VD_ or ETC subunit protein content.

## 2 MATERIALS AND METHODS

### 2.1 Ethical approval

This study was approved by the Queen's University Health Sciences & Affiliated Teaching Hospitals Research Ethics Board (HSREB; REB #6003260) and conformed to the standards set by the latest version of the *Declaration of Helsinki*. All participants provided verbal and written informed consent before participating in the study.

### 2.2 Participants

The data presented herein were obtained from 13 females and 13 males who were recruited as part of a separate investigation (registered on Open Science Framework: https://osf.io/kzvgc). Participants self‐identified as female or male. The study design, inclusion criteria and some of the participant characteristics for the males have been published elsewhere (Pacitti et al., 2024). Baseline participant characteristics are provided in Table 1.

**TABLE 1:** Participant characteristics.

Variable	Female (*n* = 13)	Male (*n* = 13)^a^
Age, years	22 ± 3	22 ± 2
Height, m	1.69 ± 0.06	1.81 ± 0.08^**^
BM, kg	64.7 ± 7.1	78.8 ± 11.4^**^
BMI, kg/m^2^	22.6 ± 2.0	24.0 ± 2.5
Absolute FFM, kg	47.1 ± 4.0	66.0 ± 7.8^***^
Body fat, %	26.9 ± 4.8	15.9 ± 3.9^***^
${\dot{V}}_{O_{2}\mathrm{peak}}$, L/min	2.7 ± 0.3	3.7 ± 0.6^***^
${\dot{V}}_{O_{2}\mathrm{peak}}$, ml/kg BM/min	41.7 ± 5.2	47.3 ± 4.5^*^
${\dot{V}}_{O_{2}\mathrm{peak}}$, mk/kg FFM/min	57.1 ± 6.5	56.3 ± 5.6

*Note*: Data are shown as the mean ± SD. Statistical significance was set at *P < *0.05.

Abbreviations: BM, body mass; BMI, body mass index; FFM, fat‐free mass; ${\dot{V}}_{O_{2}\mathrm{peak}}$, peak oxygen uptake.

^a^ Participant characteristics for males, excluding absolute FFM and body fat percentage, previously published by Pacitti et al. (2024).

^*^ *P *< 0.01, ^**^ *P *< 0.001 and ^***^ *P *< 0.0001.

### 2.3 Study design

Participants initially attended the laboratory for a baseline visit, during which anthropometric measures were recorded, and participants completed a ${\dot{V}}_{O_{2}\mathrm{peak}}$ test on a cycle ergometer (Ergomedic 874E, Monark, Varberg, Sweden). Anthropometric measures included height, weight, and body composition assessed via bioelectrical impedance analysis (TANITA Model BC‐418, TANITA, USA), as discussed previously (Pacitti et al., 2024). Exercise testing for baseline ${\dot{V}}_{O_{2}\mathrm{peak}}$ was performed using a step protocol as described previously (Pacitti et al., 2024). Briefly, participants completed a load‐free warm‐up, followed by an increase in work rate to ∼80 W for 1 min, and work rate was increased by 24 W each minute until the participant reached volitional fatigue (Pacitti et al., 2024). The ${\dot{V}}_{O_{2}\mathrm{peak}}$ was calculated as the highest 30 s average O_2_ uptake during the final stage of the protocol (Pacitti et al., 2024).

A skeletal muscle biopsy of the vastus lateralis was obtained during a separate experimental visit after the baseline visit. Participants were asked to refrain from alcohol and exercise for 24 h before each experimental visit. All experimental visits were separated by a minimum of 48 h. All skeletal muscle biopsies, mitochondrial respiration and western blotting analysis were performed at a single site (Queen's University).

### 2.4 Skeletal muscle biopsy

A skeletal muscle biopsy of the vastus lateralis was obtained in the rested, fasted state, as previously described (McGlory et al., 2019). Participants arrived at the laboratory after an overnight fast (water was consumed ad libitum). The participant lay in the supine position, and the muscle biopsy collection site was cleaned with Baxedin solution. Local freezing (2% lignocaine plus adrenaline) was injected into both the skin and the fascia before the biopsy using a Bergström needle, modified with manual suction.

### 2.5 Permeabilized skeletal muscle fibres

Immediately after the biopsy, muscle tissue allocated to mitochondrial respiration analysis was placed in ice‐cold BIOPS (pH 7.2) containing 2.77 mM CaK_2_EGTA, 0.5 mM dithiothreitol, 20 mM imidazole, 7.23 mM K_2_EGTA, 50 mM MES hydrate, 6.56 mM MgCl_2_.6H_2_O, 5.77 mM Na_2_ATP, 15 mM Na_2_PCr, and 20 mM taurine. Skeletal muscle fibre bundles were mechanically separated and weighed in duplicate, as previously described (Ferguson et al., 2024). Muscle fibre bundle masses were obtained before permeabilization with saponin. The average of the duplicate mass values was used for data normalization (wet weight). Fibre bundles were chemically permeabilized in BIOPS with 40 µg/ml saponin for 30 min at 4°C with gentle rocking on a nutator (Kuznetsov et al., 2008). The PmFBs were then washed in Buffer Z (no creatine) for 15 min at 4°C with gentle rocking on a nutator (until respiratory measurements were initiated). Buffer Z (pH 7.2) contains 5 mg/ml fatty acid‐free bovine serum albumin, 1 mM EGTA, 30 mM KCl, 10 mM KH_2_PO4, 105 mM K‐MES and 5 mM MgCl_2_.6H_2_O.

### 2.6 High‐resolution respirometry protocol

Rates of skeletal muscle mitochondrial oxygen consumption were measured using an Oroboros O2K high‐resolution respirometer (Oroboros Instruments, Innsbruck, Austria). All respiration measurements were performed in 2 mL of Buffer Z with or without 20 mM creatine (Perry et al., 2012, 2011; Ydfors et al., 2016), with stirring at 750 r.p.m. and at a temperature of 37°C. Mitochondrial respiration measurements were obtained while the chamber oxygen concentration was between 390 and 180 µM. Data were acquired every 2 s, with the uncorrected rate of mitochondrial oxygen consumption (in picomoles per second per millilitre) calculated from 40 data points.

The PmFBs were assayed in duplicate in the presence of the myosin ATPase inhibitor blebbistatin (Perry et al., 2011). 5 µM of blebbistatin (Hughes et al., 2015) was added to each chamber before PmFB insertion and chamber oxygenation. 5 mM pyruvate and 2 mM malate (PM) were added consecutively as complex I substrates. ADP titrations of 25, 100, 500, 1000, 5000, 7000, 10,000 and 12,000 µM allowed for the characterization of respiration at both physiological (Howlett et al., 1998; Perry et al., 2008; Philips et al., 1996) and maximal ADP ranges (PMD). ADP titrations of 10 000 and 12 000 µM were not included in the 20 mM creatine condition because maximal rates of respiration were obtained at lower ADP concentrations. Next, 10 mM glutamate was added to the chamber to examine maximal complex I‐supported, ADP‐stimulated mitochondrial respiration (PMDG). Subsequently, 10 µM cytochrome *c* was added as a quality control test to examine mitochondrial membrane integrity. If a fibre bundle had a cytochrome *c* response showing an increase in the rate of respiration above 15% and low maximal respiration rates, the fibre bundle would be excluded. No fibre bundles were excluded based on cytochrome *c* responses in this investigation. Finally, 20 mM succinate was added to examine maximal complex I+II‐supported, ADP‐stimulated mitochondrial respiration (PMDGS).

Data corrected for bundle wet weight are shown in picomoles per second per milligram wet weight, and data normalized to total mito_VD_ and ETC subunit protein content are expressed as picomoles/second/milligram wet weight/total mito_VD_ and picomoles/second/milligram wet weight/ETC subunit protein content, respectively. Mitochondrial creatine sensitivity index values were calculated as the rate of mitochondrial respiration in the 20 mM creatine Buffer Z condition divided by the rate of mitochondrial respiration in the non‐creatine Buffer Z condition at a given ADP concentration (25–500 µM; Delfinis et al., 2022). Mitochondrial respiration data were not obtained for one female and one male participant. Rates of respiration for two male participants that were obtained on the same experimental day were excluded from the analysis owing to low responsiveness to ADP titrations (i.e., the samples did not respire despite the increased stimulus provided by increasing concentrations of ADP) (Bellissimo et al., 2023).

### 2.7 Transmission electron microscopy

Samples for transmission electron microscopy (TEM) were prepared by a single, blinded technician in the Queen's CardioPulmonary Unit at Queen's University. Skeletal muscle samples were fixed with 2.5% glutaraldehyde in 0.1 M sodium cacodylate buffer (pH 7.4). Samples were subsequently rinsed five times in 0.1 M sodium cacodylate buffer. After rinsing, fibres were secondarily fixed in 1% osmium tetroxide in 0.1 M sodium cacodylate buffer. After fixation, fibres were rinsed five times using double distilled water. Fibres were then dehydrated in ascending ethanol concentrations and infiltrated with propylene oxide and Epon. Finally, fibres were embedded in 100% Epon at 65°C.

**FIGURE 1:**
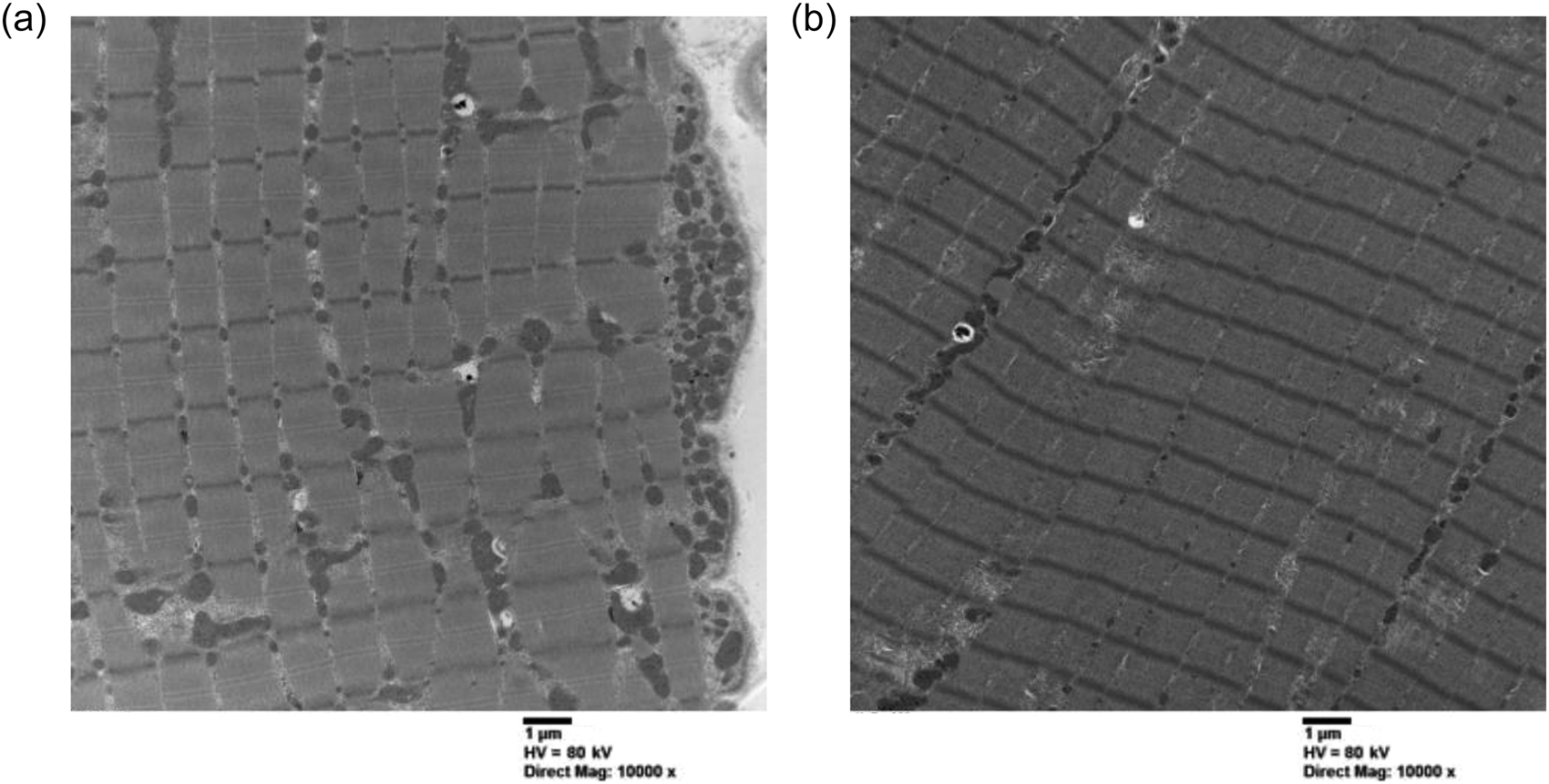
Representative transmission electron micrographs for SS (a) and IMF mito_VD_ (b). Abbreviations: IMF, intermyofibrillar; mito_VD_, mitochondrial volume density; SS, subsarcolemmal.

Thin sections were cut on a Leica UCT ultramicrotome (Leica Microsystems, Vienna, Austria) and picked up onto copper grids and stained with uranyl acetate and lead citrate. A transmission electron microscope (JEOL 1200 EX TEMSCAN) was used to view the grids at ×10 000 magnification. Images were obtained by a single, blinded technician in the Canadian Centre for Electron Microscopy (CCEM) at McMaster University. Images were acquired with an AMT 4‐megapixel digital camera (Advanced Microscopy Techniques). Thirty‐two images were obtained per skeletal muscle sample. Specifically, two images from the IMF and two images from the SS regions for eight fibres were obtained in a random manner to examine IMF, SS and total mito_VD_ (Skelly et al., 2023). Representative TEM images are shown in Figure 1.

Mito_VD_ was determined using point counting (ImageJ, v.1.53t, US National Institutes of Health, Bethesda, MD, USA; Broskey et al., 2013). Briefly, a 500 nm × 500 nm (0.25 µm^2^) grid was overlaid on each micrograph (Broskey et al., 2013). The ratio of points that touched IMF or SS mitochondria to the number of points that touched muscle fibre area was used to determine the population‐specific mito_VD_ (Meinild‐Lundby et al., 2018; Skelly et al., 2023). Total mito_VD_ was defined as the sum of IMF and SS mito_VD_ (Schytz et al., 2024). The SS mitochondria were classified as mitochondria that were not separated from the sarcolemma by myofibrils (Skelly et al., 2023). Given that SS and IMF images were acquired in a random manner, some points on the image did not belong to the muscle specimen, and these points were excluded from the analysis (Schytz et al., 2024). TEM samples were not collected for one female and one male participant, and images for two female and one male participant were excluded owing to poor image quality, yielding a final sample size of 11 and 10 for males and females, respectively.

All TEM images were analysed by a single, blinded member of the research team (C.J.P.). Intra‐rater reliability was determined 8 weeks apart using four random images from each participant (Stokes et al., 2021). Intraclass correlation coefficient values were calculated using a two‐way mixed‐effects model, absolute agreement, single rater/measurement (Islam et al., 2019; Stokes et al., 2021; Koo and Li, 2016). The intra‐rater reliability analysis yielded an intraclass correlation value of 0.977, indicating excellent intra‐rater reliability (Koo and Li, 2016).

### 2.8 Western blotting

Skeletal muscle samples were homogenized in ice‐cold RIPA buffer (Thermo Fisher Scientific, Waltham, MA, USA). The total protein concentration of the skeletal muscle sample was determined using a colorimetric bicinchoninic acid assay (Thermo Fisher Scientific, Waltham, MA, USA). Equal amounts of protein (11 µg) from each sample homogenate were loaded into 4%–15% Criterion TGX Stain‐free protein gels (Bio‐Rad, Mississauga, ON, Canada) to quantify ETC subunit protein content. Each sample was assayed in duplicate, and an unstained protein ladder (Bio‐Rad) and calibration curve were run on each gel. Gels were run at 200 V for 45 min prior to transfer to a low‐fluorescence polyvinylidene difluoride membrane. Ultraviolet activation of the gel was performed (ChemiDoc MP Imaging System; Bio‐Rad) prior to rapid transfer for all gels using the Transblot Turbo System (Bio‐Rad). Membranes were blocked for 1.5 h at room temperature in 5% bovine serum albumin and incubated in the primary antibody (OXPHOS cocktail; 1:1000; ab110413; Abcam, Cambridge, UK) for 12 h at 4°C. Membranes were incubated in the anti‐mouse IgG conjugated with horseradish peroxidase secondary antibody (1:5000; AP308P; MilliporeSigma, Oakville, ON, Canada) for 1 h at room temperature following three washes with 1× Tris‐buffered saline and Tween20. Signals were detected via chemiluminescence using enhanced chemiluminescence (ECL; Bio‐Rad) substrate on the ChemiDoc MP Imaging System (Bio‐Rad). Total lane protein density from the membrane post‐transfer was analysed using Image Lab v.6.1 software (Bio‐Rad). Protein expression of complex I subunit NDUFB8 (20 kDa), complex II SDHB (30 kDa), complex III core protein 2 (UQCRC2; 48 kDa), complex IV subunit 1 (MTCO1; 40 kDA) and complex V alpha subunit (ATP5A; 55 kDa) was analysed using Image Lab v.6.1 software (Bio‐Rad). Total lane protein densities and band densities for each ETC subunit were adjusted using calibration curves. Band densities for each ETC subunit were normalized to the total lane protein loaded for a given lane (Caswell et al., 2024; McDougall et al., 2023). Total ETC subunit protein content was calculated as the sum of the protein content for each ETC complex subunit. Western blot data were obtained for 13 females and 13 males.

### 2.9 Statistical analysis

Rates of mass‐specific and intrinsic respiration, in addition to creatine sensitivity, were analysed using a mixed‐effects model, with sex (female or male) and treatment (substrate or ADP concentration) as factors. All data analysed using a mixed‐effects model were assessed for normality of the residuals. If the omnibus statistical tests revealed significant interaction effects, additional *post hoc* analysis using the Bonferroni correction was performed. Participant baseline characteristics, mito_VD_ and ETC subunit protein content data were assessed for normality using the D'Agostino–Pearson omnibus K2 test and analysed using Student's unpaired *t*‐test. Pearson's correlations were used to assess the relationship between ${\dot{V}}_{O_{2}\mathrm{peak}}$ normalized to FFM and total, IMF and SS mito_VD_, in addition to ETC subunit protein content when data were collapsed across females and males. Michaelis–Menten modelling was performed using GraphPad Prism v.10.1.1 (GraphPad Software, San Diego, CA, USA). Apparent *K*_m_ (*K*_mapp_) values were calculated relative to the highest maximal respiration rate obtained (defined as *V*_max_) and analysed using a Student's unpaired *t*‐test. Data are presented as box and whisker plots in figures or the mean ± standard deviation (SD) in tables and the main text. 95% confidence intervals are also presented in brackets in the main text. Statistical analysis was performed using GraphPad Prism v.10.1.1 (GraphPad Software). Statistical significance was set at *P < *0.05.

## 3 RESULTS

### 3.1 Mass‐specific mitochondrial respiration

**FIGURE 2:**
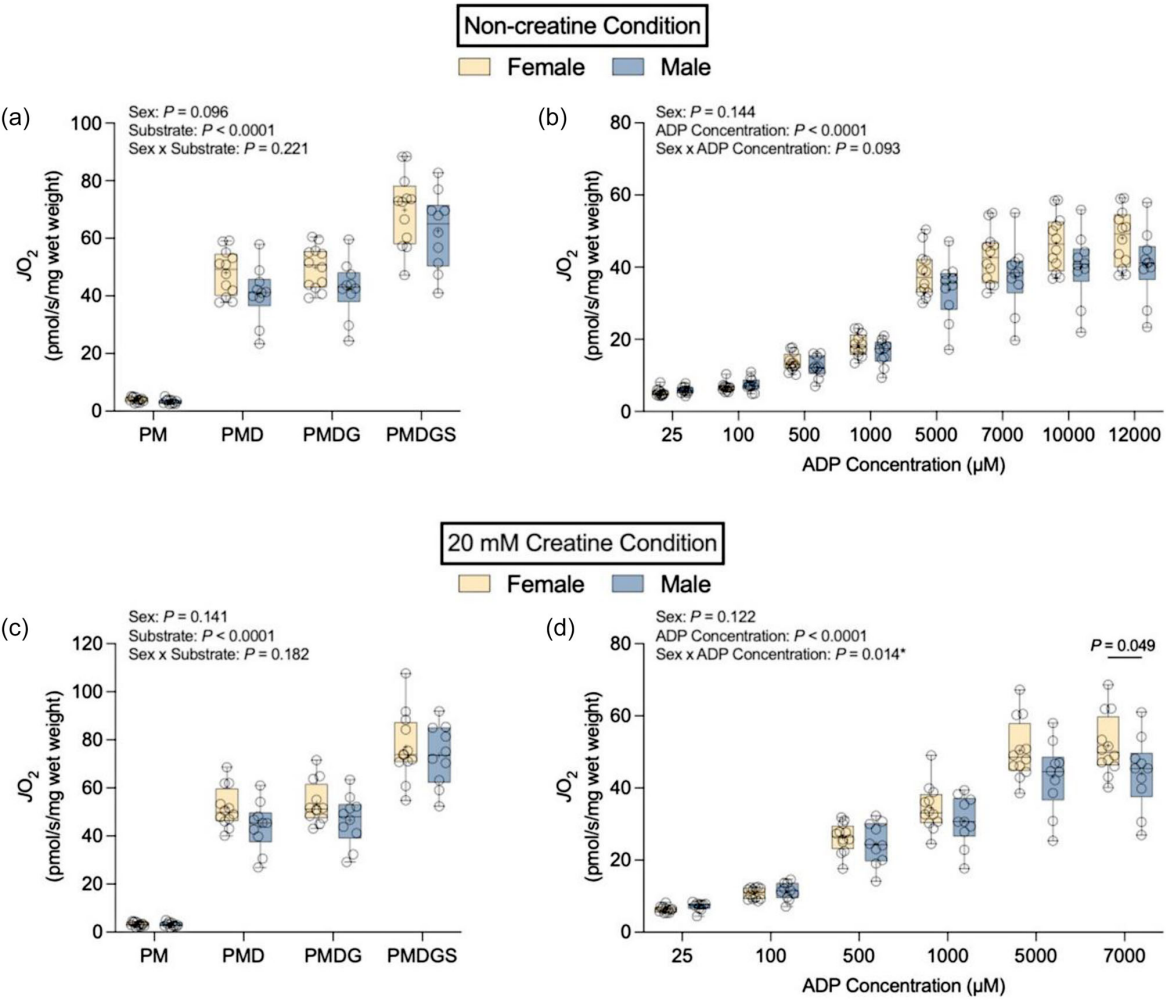
Mass‐specific mitochondrial respiration in both non‐creatine (a, b) and 20 mM creatine (c, d) conditions. (a) Rates of pyruvate and malate (PM)‐supported, maximal ADP‐stimulated (PMD), maximal complex I‐supported ADP‐stimulated (PMDG) and maximal complex I+II‐supported ADP‐stimulated (PMDGS) respiration in the non‐creatine condition. (b) Rates of PM‐supported, ADP‐stimulated respiration in the non‐creatine condition. (c) Rates of PM, PMD, PMDG and PMDGS‐ supported respiration in the 20 mM creatine condition. (d) Rates of PM‐supported, ADP‐stimulated respiration in the 20 mM creatine condition. Data are shown as box and whisker plots, with the mean shown as a plus sign and the median as a horizontal line. Individual data points are shown as circles (female, *n* = 12; male, *n* = 10).

There was no statistically significant sex × substrate (PM, PMD, PMDG and PMDGS) interaction in the non‐creatine (*P *= 0.221; Figure 2a) or 20 mM creatine conditions (*P *= 0.182; Figure 2c). There was no effect of sex (*P *= 0.096 non‐creatine condition; *P *= 0.141 20 mM creatine condition), but there was a statistically significant effect of substrate (*P *< 0.0001) in both the non‐creatine and 20 mM creatine conditions. In the non‐creatine condition, the sex × ADP concentration interaction for rates of respiration stimulated by a range of ADP concentrations from physiological (25 µM) to maximal and supraphysiological (12 000 µM) was not significant (*P *= 0.093; Figure 2b). There was no effect of sex (*P *= 0.144), but there was a significant effect of ADP concentration (*P *< 0.0001). There was a statistically significant sex × ADP concentration interaction (*P *= 0.014; Figure 2d) and a significant effect of ADP concentration (*P *< 0.0001), but no significant effect of sex (*P *= 0.122) in the 20 mM creatine condition. *Post hoc* analysis revealed higher rates of respiration stimulated by 7000 µM ADP in females compared with males [*P *= 0.049; female, 51.75 ± 8.44 pmol/s/mg wet weight; male, 44.10 ± 10.06 pmol/s/mg wet weight; female vs. male, 7.65 pmol/s/mg wet weight (0.05; 15.24)]. We did not detect a statistically significant difference in *K*_mapp_ between females and males in the presence [female, 676 ± 137 µM ADP; male, 608 ± 107 µM ADP; female vs. male,  +68 µM ADP (−43; 179); *P *= 0.215] or absence [female, 2628 ± 650 µM ADP; male, 2611 ± 556 µM ADP; female vs. male, +17 µM ADP (−527; 561); *P *= 0.950] of 20 mM creatine (data not shown). These data were analysed with absolute respiration rates (*R* ^2^ range 0.800–0.898 for the Michaelis–Menten curve), but similar conclusions were obtained when using data expressed as a percentage of maximal respiration (*R* ^2^ range 0.943–0.979 for the Michaelis‐Menten curve; data not shown).

### 3.2 Creatine sensitivity

**FIGURE 3:**
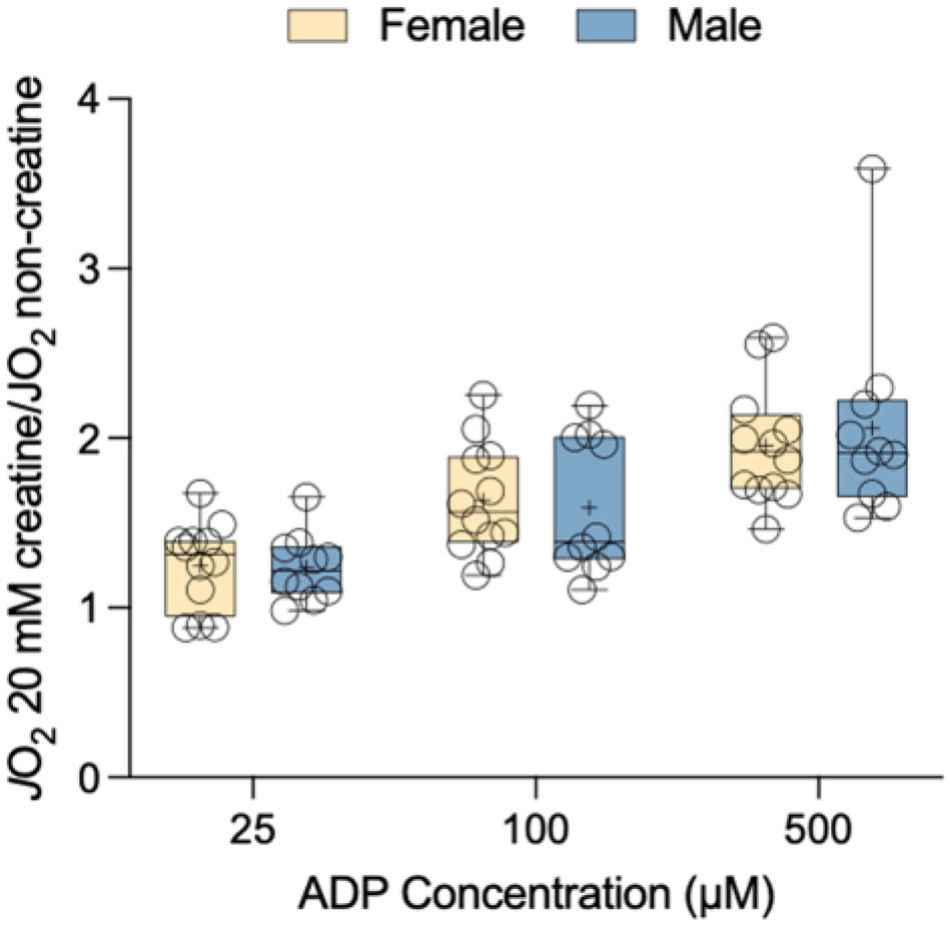
Creatine sensitivity index values for rates of mitochondrial respiration supported by 25–500 µM ADP. Data are presented as box and whisker plots, with the mean and median denoted as a plus sign and horizontal line, respectively. Individual data points are shown as circles (female, *n* = 12; male, *n* = 10).

We did not detect a statistically significant ADP concentration × sex interaction (*P *= 0.731) for creatine sensitivity values across a range of ADP concentrations (Figure 3). There was no statistically significant effect of sex (*P *= 0.882), but there was a significant effect of ADP concentration (*P *< 0.0001).

### 3.3 Total and population‐specific mito_VD_

**FIGURE 4:**
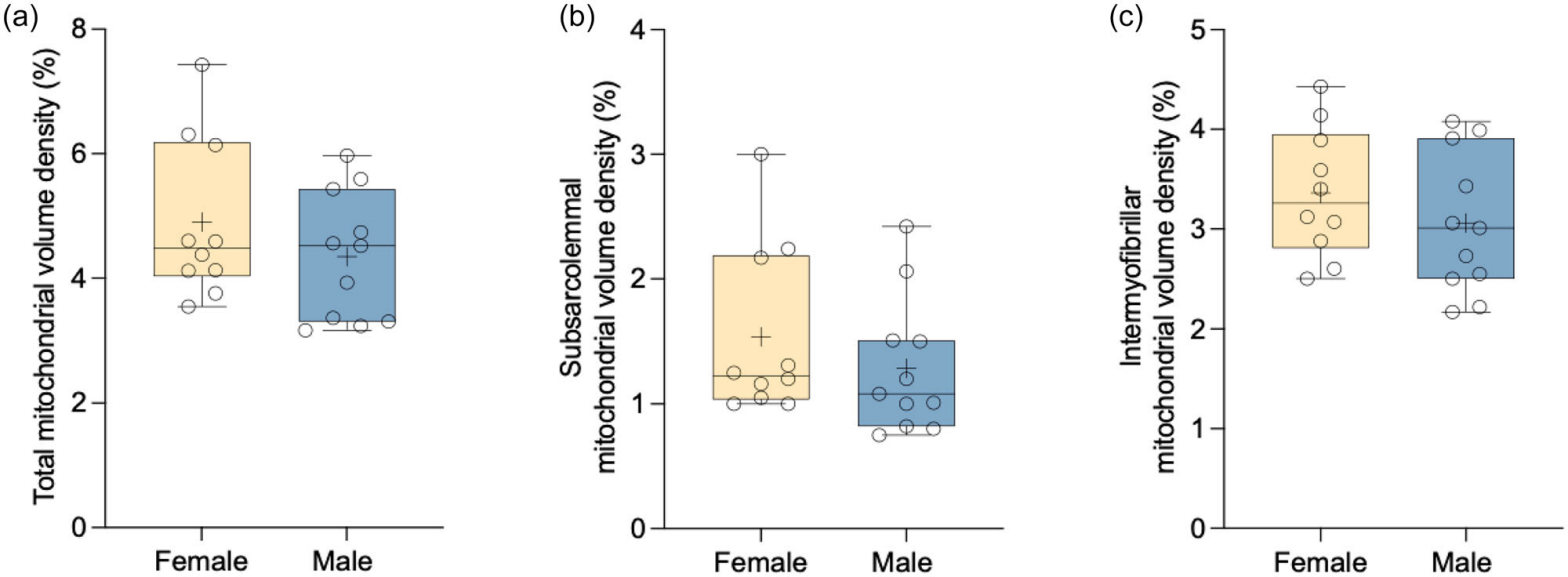
Total (a), SS (b) and IMF (c) mitochondrial volume density. Data are presented as box and whisker plots, with the mean and median denoted by a plus sign and horizontal line, respectively. Individual data points are shown as circles (female, *n* = 10; male, *n* = 11). Abbreviations: IMF, intermyofibrillar; SS, subsarcolemmal.

There were no statistically significant differences between females and males for total (*P *= 0.285; Figure 4a), SS (*P *= 0.360; Figure 4b) or IMF (*P *= 0.319; Figure 4c) mito_VD_.

**FIGURE 5:**
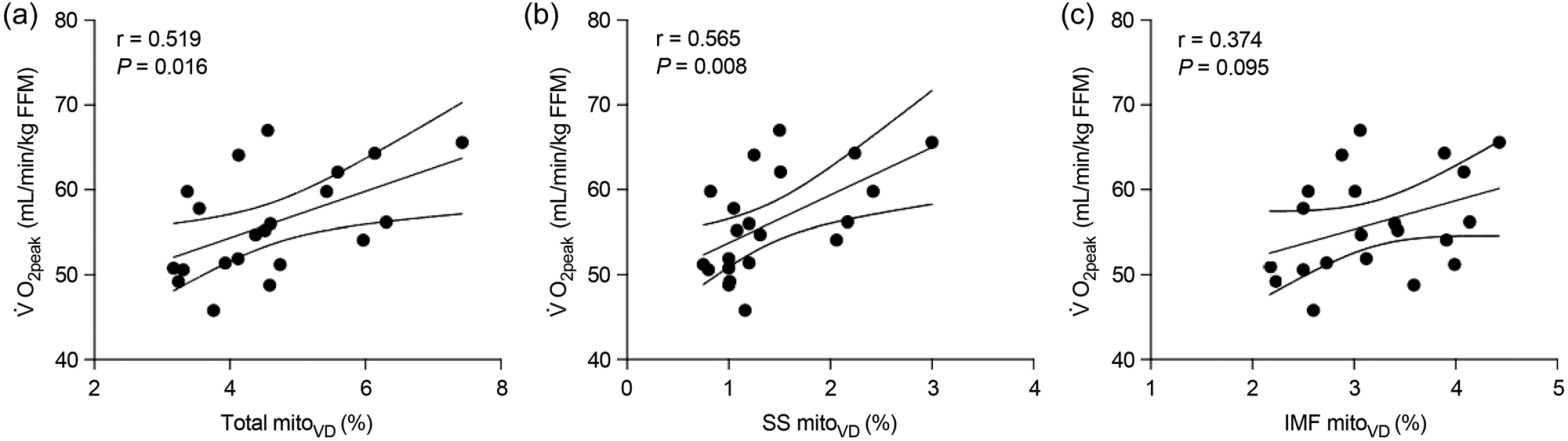
Pearson's correlations between ${\dot{V}}_{O_{2}\mathrm{peak}}$ normalized to FFM and total (a), SS (b) and IMF (c) mito_VD_. Data have been collapsed across both female and male participants (female, *n* = 10; male, *n* = 11). The continuous line represents the line of best fit, and the dotted lines represent the 95% confidence intervals. Abbreviations: FFM, fat‐free mass; IMF, intermyofibrillar; mito_VD_, mitochondrial volume density; SS, subsarcolemmal; ${\dot{V}}_{O_{2}\mathrm{peak}}$, peak oxygen uptake.

Pearson's correlations revealed statistically significant moderate correlations between ${\dot{V}}_{O_{2}\mathrm{peak}}$ normalized to FFM and total [*r* = 0.519 (0.113; 0.777); *P *= 0.016; Figure 5a] and SS [*r* = 0.565 (0.177; 0.801); *P *= 0.008; Figure 5b] mito_VD_. We observed a weak to moderate and not statistically significant correlation between ${\dot{V}}_{O_{2}\mathrm{peak}}$ normalized to FFM and IMF mito_VD_ [*r* = 0.374 (−0.068; 0.694); *P *= 0.095; Figure 5c].

### 3.4 Electron transport chain subunit protein content

**FIGURE 6:**
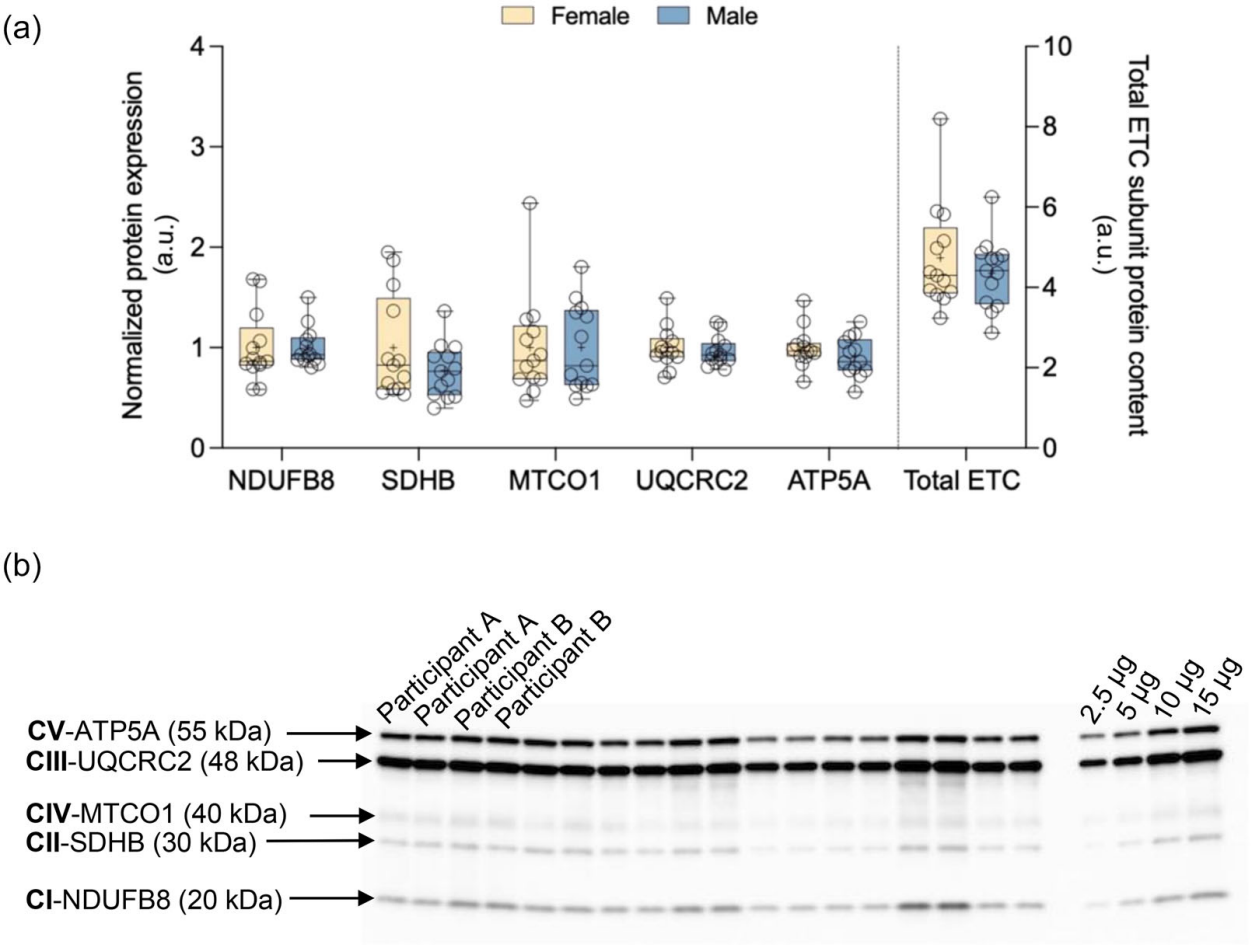
(a) ETC subunit protein content. Protein expression of complex I subunit NDUFB8 (20 kDa), complex II subunit SDHB (30 kDa), complex IV subunit 1 (MTCO1; 40 kDA), complex III core protein 2 (UQCRC2; 48 kDa) and complex V alpha subunit (ATP5A; 55 kDa) is shown. Data for individual ETC complex subunits (left *y*‐axis) and the sum (right *y*‐axis) are shown. Data are presented as box and whisker plots, with the mean and median denoted as a plus sign and horizontal line, respectively. Data are shown normalized to average of female data for each subunit. Individual data points are shown as circles (female, *n* = 13; male, *n* = 13). (b) Representative blot for ETC subunit protein content. Abbreviation: ETC, Electron transport chain.

Our results did not demonstrate a statistically significant difference in total ETC subunit protein content between females and males (*P *= 0.403). There were also no statistically significant differences observed for individual ETC subunit protein content (NDUFB8, *P *= 0.978; SDHB, *P *= 0.170; MTCO1, *P *= 0.992; UQCRC2, *P *= 0.581; ATP5A, *P *= 0.249; Figure 6). We observed a weak and not statistically significant correlation between ${\dot{V}}_{O_{2}\mathrm{peak}}$ normalized to FFM and total ETC subunit protein content [*r* = 0.218 (−0.185, 0.558); *P *= 0.286; data not shown].

### 3.5 Intrinsic mitochondrial respiration

**FIGURE 7:**
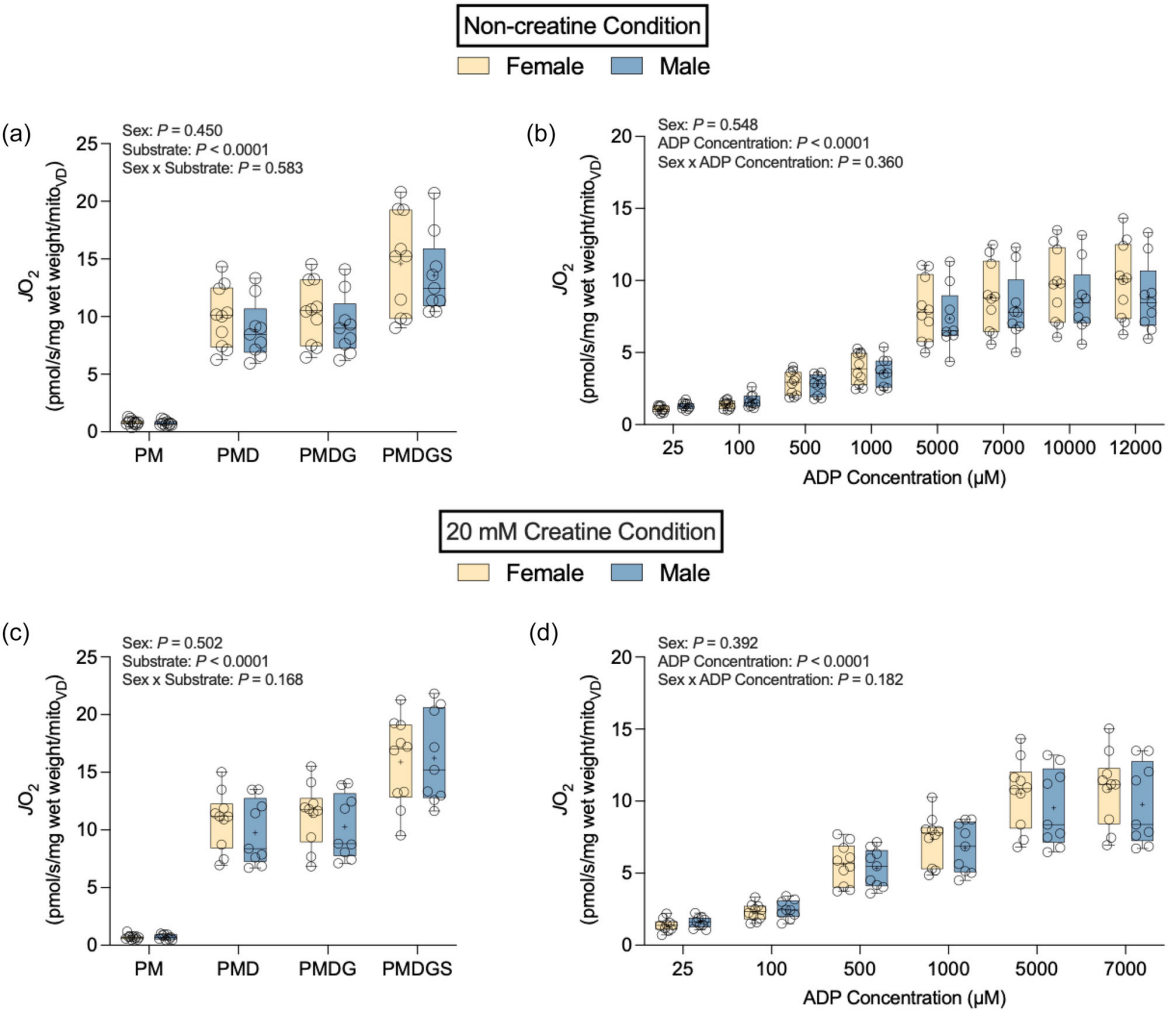
Intrinsic rates of respiration using total mito_VD_ in both non‐creatine (a, b) and 20 mM creatine (c, d) conditions. (a) Rates of PM‐supported, maximal ADP‐stimulated (PMD), maximal complex I‐supported ADP‐stimulated (PMDG) and maximal complex I+II‐supported ADP‐ stimulated (PMDGS) respiration in the non‐creatine condition. (b) Rates of PM‐supported, ADP‐stimulated respiration in the non‐creatine condition. (c) Rates of PM, PMD, PMDG and PMDGS‐supported respiration in the 20 mM creatine condition. (d) Rates of PM‐supported, ADP‐stimulated respiration in the 20 mM creatine condition. Data are shown as box and whisker plots, with the mean shown as a plus sign and the median as a horizontal line. Individual data points are shown as circles (female, *n* = 10; male, *n* = 9). Abbreviations: mitoVD, mitochondrial volume density; PM, pyruvate and malate.

There were no statistically significant sex × substrate interaction effects in the non‐creatine (*P *= 0.583; Figure 7a) or 20 mM creatine (*P *= 0.168; Figure 7c) condition when analysing rates of mitochondrial respiration normalized to total mito_VD_. There was no significant effect of sex (*P *= 0.450) but there was an effect of substrate (*P *< 0.0001) in the non‐creatine condition. Likewise, there was no significant effect of sex (*P *= 0.502) but there was an effect of substrate (*P *< 0.0001) in the 20 mM creatine condition. We did not detect a statistically significant sex ×ADP concentration interaction in the non‐ creatine (*P *= 0.360; Figure 7b) or 20 mM creatine condition (*P *= 0.182; Figure 7d). There were no statistically significant effects of sex in the non‐creatine (*P *= 0.548) or 20 mM creatine (*P *= 0.392) condition, but there was a statistically significant effect of ADP concentration (*P *< 0.0001) in both the non‐creatine and 20 mM creatine conditions.

**FIGURE 8:**
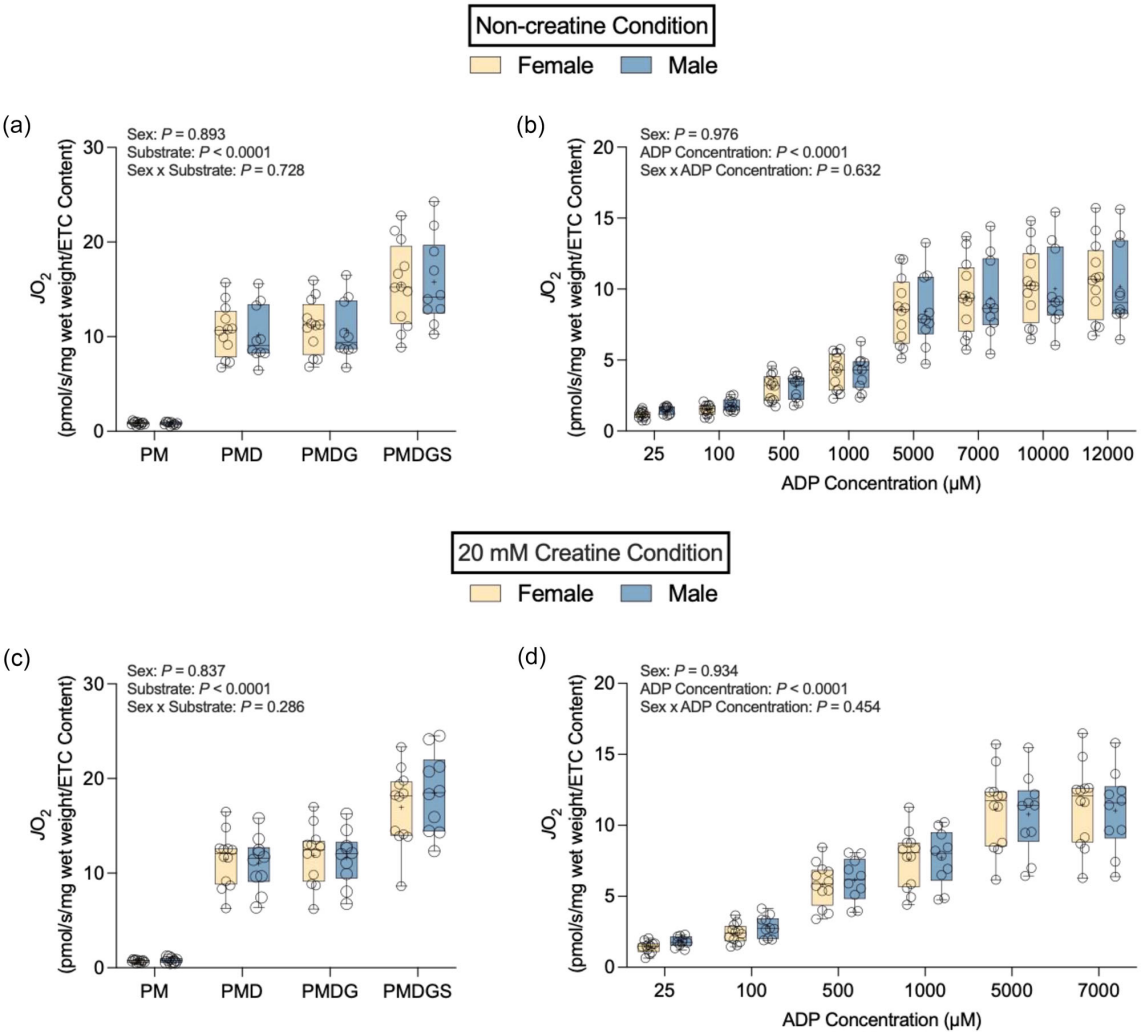
Intrinsic rates of respiration using total ETC subunit protein content as a biomarker of mitochondrial content. (a) Rates of PM‐supported, maximal ADP‐stimulated (PMD), maximal complex I‐supported ADP‐stimulated (PMDG) and maximal complex I+II‐supported ADP‐stimulated (PMDGS) respiration in the non‐creatine condition. (b) Rates of PM‐supported, ADP‐stimulated respiration in the non‐creatine condition. (c) Rates of PM, PMD, PMDG and PMDGS‐supported respiration in the 20 mM creatine condition. (d) Rates of PM‐supported, ADP‐stimulated respiration in the 20 mM creatine condition. Data are shown as box and whisker plots, with the mean shown as a plus sign and the median as a horizontal line. Individual data points are shown as circles (female, *n* = 12; male, *n* = 10). Abbreviations: ETC, electron transport chain; PM, pyruvate and malate.

Normalizing rates of mass‐specific respiration to total ETC subunit protein content did not yield statistically significant differences in rates of mitochondrial respiration. The sex × substrate interaction (*P *= 0.728; Figure 8a) and effect of sex (*P *= 0.893) were not statistically significant in the non‐creatine condition. We also did not detect a significant sex × substrate interaction (*P *= 0.286; Figure 8c) or effect of sex (*P *= 0.837) in the 20 mM creatine condition. There was a significant effect of substrate in both the non‐creatine and 20 mM creatine conditions (*P *< 0.0001). There were no statistically significant sex × ADP concentration interactions in the non‐creatine (*P *= 0.632; Figure 8b) and 20 mM creatine conditions (*P *= 0.454; Figure 8d). The effects of sex in the non‐creatine and 20 mM creatine conditions were also not significant (*P *= 0.976 and *P *= 0.934, respectively). In both the non‐creatine and 20 mM creatine conditions there was a significant effect of ADP concentration (*P *< 0.0001).

## 4 DISCUSSION

The primary aim of the present study was to examine whether biological sex impacts mass‐specific and intrinsic complex I‐ and complex I+II‐supported, ADP‐ stimulated mitochondrial respiration in PmFBs from females and males matched for ${\dot{V}}_{O_{2}\mathrm{peak}}$ normalized to FFM. We also sought to determine whether females and males matched for ${\dot{V}}_{O_{2}\mathrm{peak}}$ normalized to FFM exhibit different respiration rates stimulated by creatine‐independent ADP diffusion and creatine‐dependent phosphate shuttling. We provide new data by showing an overall lack of sex‐specific differences in: (1) rates of PM‐supported, ADP‐stimulated and maximal complex I‐ and complex I+II‐supported, ADP‐stimulated mass‐specific respiration in the presence and absence of creatine; (2) total, IMF and SS mito_VD_ and ETC subunit protein content; and (3) rates of PM‐supported, ADP‐stimulated and maximal complex I‐ and complex I+II‐supported, ADP‐stimulated intrinsic respiration in the presence and absence of creatine.

Collectively, our data suggest a lack of sex‐based differences in mass‐specific respiration rates obtained in the absence of creatine. This observation supports previous work showing no statistically significant differences in rates of maximal complex I‐ and complex I+II‐supported, ADP‐stimulated mass‐specific respiration between females and males in the absence of creatine in the respiration media (Miotto et al., 2018; Schytz et al., 2024). Others have also reported that females and males matched for ${\dot{V}}_{O_{2}\mathrm{peak}}$ normalized to total body mass do not exhibit differences in maximal mass‐specific state 3 respiration and oxidative phosphorylation capacity in the absence of creatine (Montero et al., 2018). However, a unique finding of the present study is that the addition of creatine to the respiration media resulted in higher PM‐supported, ADP‐stimulated mass‐specific respiration in females compared with males but only at a concentration of 7000 µM ADP. It is important to note that this concentration of ADP is supraphysiological, and the higher rate of mitochondrial respiration in females compared with males was abolished when respiration data were normalized to total mito_VD_ and ETC subunit protein content. Although the physiological relevance of this finding is unclear, the higher rate of mass‐specific respiration in females at 7000 µM ADP could have been driven by slight, but undetectable, differences in mitochondrial content.

In addition to examining maximal rates of PM‐supported, ADP‐stimulated mitochondrial respiration, we modelled in vivo conditions by including physiological ADP concentrations in our in vitro protocol. Specifically, we examined rates of respiration stimulated by resting (25 µM) and exercising (100 µM) ADP concentrations (Howlett et al., 1998; Perry et al., 2008; Phillips et al., 1996). We identified no differences in respiration rates stimulated by these concentrations of ADP, both in the presence and absence of a physiological concentration of creatine (Tonkonogi et al., 1998; Ydfors et al., 2016). In contrast, others have shown higher rates of mass‐specific PM‐supported respiration stimulated by 100 µM ADP in males versus females in the absence of creatine (Miotto et al., 2018). Given that ${\dot{V}}_{O_{2}\mathrm{peak}}$ was not measured in the study by Miotto et al. (2018) it is plausible that differences in participant aerobic fitness might explain the higher absolute mitochondrial respiration rates in males relative to females. However, no sex differences in maximal mitochondrial respiration or ETC subunit protein content were observed in that study (Miotto et al., 2018), suggesting that any differences in ${\dot{V}}_{O_{2}\mathrm{peak}}$ might have been minor. It is worthwhile noting that intrinsic rates of respiration were not analysed in the study by Miotto et al. (2018), thus it is unknown whether this difference would have persisted when mass‐specific rates of respiration were normalized to a marker of mitochondrial content. Nonetheless, our results imply that matching participants for ${\dot{V}}_{O_{2}\mathrm{peak}}$ normalized to FFM mitigates sex‐based differences in aerobic fitness and therefore any potential differences in submaximal ADP‐stimulated respiration between females and males.

We sought to determine whether females and males matched for aerobic fitness exhibit different rates of intrinsic mitochondrial respiration. Our results demonstrate an overall lack of effect of sex on rates of intrinsic PM‐supported, ADP‐stimulated respiration or on maximal complex I‐ and complex I+II‐supported, ADP‐stimulated respiration. We also show no sex‐based differences in intrinsic mitochondrial respiratory capacity in the presence and absence of creatine when mass‐specific respiration rates are normalized to total mito_VD_ and ETC subunit protein content. Our findings corroborate those of Montero et al. (2018), whereby differences in mass‐specific respiration between females and males were abolished when rates of respiration were adjusted for total mito_VD_. Our findings also agree with the analysis by Schytz et al. (2024), who did not observe sex‐based differences in mitochondrial‐ and cristae‐specific respiration rates in active females and males, and Thompson et al. (2013), who found no differences in rates of intrinsic respiration in PmFBs from gastrocnemius muscle from middle‐aged females and males. However, our findings contrast with those of Cardinale and colleagues (2018), who found higher intrinsic mitochondrial respiration rates in females versus males matched for ${\dot{V}}_{O_{2}\mathrm{peak}}$ normalized to total body mass in isolated mitochondria from vastus lateralis muscle. It could be speculated that the use of different mitochondrial preparations (i.e., isolated mitochondria vs. PmFBs) might influence observations of sex‐based differences in rates of respiration. Future work that directly measures rates of mitochondrial respiration in isolated mitochondria and PmFBs from females and males matched for aerobic fitness is required to substantiate this speculative hypothesis.

Rapid phosphate shuttling between the mitochondrial intermembrane space and cytosol via mitochondrial and cytosolic creatine kinases enhances ADP‐stimulated respiration, particularly at submaximal ADP concentrations (Delfinis et al., 2022; Schlattner et al., 2018; Walliman et al., 2011). Given previous reports of potential sex differences in submaximal respiration between females and males (Miotto et al., 2018), we analysed creatine sensitivity index values to explore how creatine‐dependent phosphate shuttling can enhance respiration beyond the level elicited by ADP diffusion alone (Delfinis et al., 2022). Consistent with our overall findings, we did not detect an effect of sex on creatine sensitivity. This finding would suggest that creatine‐dependent phosphate shuttling stimulates rates of mitochondrial respiration to the same degree in females and males matched for aerobic fitness.

Skeletal muscle mitochondria reside in two distinct subpopulations, the IMF and the SS, which display key differences with respect to respiratory function, proteome and morphology (Cogswell et al., 1993; Ferreira et al., 2010; Picard et al., 2013). In the present study, we did not detect an effect of biological sex on total, IMF, or SS mito_VD_ assessed via gold‐standard TEM, or in ETC subunit protein content, which is a biomarker of mitochondrial content. Our results are consistent with previous reports showing no differences in mitochondrial area (Tarnopolsky et al., 2007), mitochondrial protein content (Caswell et al., 2024), and ETC subunit protein content (McDougall et al., 2023) between females and males matched for ${\dot{V}}_{O_{2}\mathrm{peak}}$ normalized to FFM. However, a limitation of the PmFB approach is the inability to delineate between IMF‐ and SS‐specific mitochondrial respiration. Thus, future research examining differences in IMF and SS respiratory capacity in isolated mitochondria from females and males matched for ${\dot{V}}_{O_{2}\mathrm{peak}}$ normalized to FFM would add to our findings.

Although the present investigation has several strengths, including analysis of rates of mass‐specific and intrinsic respiration, both with and without creatine, matching females and males for aerobic fitness, and the use of gold‐standard methodologies such as TEM, there are some limitations that should be addressed. First, the results from this study should be interpreted within the context of healthy young adults, and we are unable to demonstrate whether differences in intrinsic mitochondrial respiration would be apparent in older females and males matched for aerobic fitness or in diseased states. Second, it is important to acknowledge that bioelectrical impedance analysis is a surrogate measure of skeletal muscle mass. Finally, we did not address sex‐specific differences in rates of respiration supported by different substrates, such as fatty acids or reactive oxygen species emissions. Previous work suggests that females might exhibit greater sensitivity to malonyl‐CoA (Miotto et al., 2018) and rates of mass‐specific respiration supported by fatty acid oxidation that are higher than (Montero et al., 2018) or similar (Miotto et al., 2018) to males, whereas males may exhibit higher reactive oxygen species emissions than females in skeletal muscle (Junker et al., 2022). Thus, future studies are required to address whether rates of mitochondrial respiration supported by fatty acid oxidation and reactive oxygen species emissions differ between females and males matched for aerobic fitness.

## 5 CONCLUSION

Our results show an overall lack of sex‐based differences in rates of creatine‐independent mass‐specific and intrinsic PM‐supported, ADP‐stimulated respiration, as well as maximal complex I‐ and complex I+II‐supported, ADP‐stimulated respiration. We demonstrate an overall lack of differences in total, IMF and SS mito_VD_, and in ETC subunit protein content between females and males matched for ${\dot{V}}_{O_{2}\mathrm{peak}}$ normalized to FFM. We also present an initial report indicating no statistically significant differences in rates of intrinsic mitochondrial respiration supported by creatine‐dependent phosphate shuttling in females and males matched for ${\dot{V}}_{O_{2}\mathrm{peak}}$ normalized to FFM, which is a topic that has previously been largely unexplored. Our data will inform the use of mixed‐sex participant cohorts for future study designs and guide the interpretation of sex‐specific responses to various interventions.

## AUTHOR CONTRIBUTIONS

Emily J. Ferguson, Lauren J. Pacitti, Brendon J. Gurd and Chris McGlory contributed to the conception and design of the experiments. All authors contributed to the data collection, analyses, and/or data interpretation. Emily J. Ferguson and Chris McGlory drafted the manuscript, and all authors edited and/or revised the manuscript critically for important intellectual content. The final version of the manuscript was approved by all authors and all authors agree to be accountable for all aspects of the work in ensuring that questions related to the accuracy or integrity of any part of the work are appropriately investigated and resolved. All persons included as authors qualify for authorship, and all those who qualify for authorship are listed.

## CONFLICT OF INTEREST

None declared.

## Data Availability

The data that support the findings of this study are available from the corresponding author upon reasonable request.
